# Kynurenine Pathway—An Underestimated Factor Modulating Innate Immunity in Sepsis-Induced Acute Kidney Injury?

**DOI:** 10.3390/cells11162604

**Published:** 2022-08-21

**Authors:** Anna Krupa, Mikolaj M. Krupa, Krystyna Pawlak

**Affiliations:** 1 Department of Internal Medicine and Metabolic Diseases, Medical University of Bialystok, M. Sklodowskiej-Curie 24A, 15-276 Bialystok, Poland; 2 Department of Monitored Pharmacotherapy, Medical University of Bialystok, Mickiewicza 2C, 15-222 Bialystok, Poland

**Keywords:** kynurenine pathway (KP), innate immunity, immunosuppression, sepsis-induced acute kidney injury (SAKI)

## Abstract

Sepsis is a life-threatening organ dysfunction caused by a dysregulated host response to infection, and it accounts for about half of the cases of acute kidney injury (AKI). Although sepsis is the most frequent cause of AKI in critically ill patients, its pathophysiological mechanisms are not well understood. Sepsis has the ability to modulate the function of cells belonging to the innate immune system. Increased activity of indoleamine 2,3-dioxygenase 1 (IDO1) and production of kynurenines are the major metabolic pathways utilized by innate immunity cells to maintain immunological tolerance. The activation of the kynurenine pathway (KP) plays a dual role in sepsis—in the early stage, the induction of IDO1 elicits strong proinflammatory effects that may lead to tissue damage and septic shock. Afterwards, depletion of tryptophan and production of kynurenines contribute to the development of immunosuppression that may cause the inability to overpower opportunistic infections. The presented review provides available data on the various interdependencies between elements of innate immunity and sepsis-induced AKI (SAKI) with particular emphasis on the immunomodulatory significance of KP in the above processes. We believe that KP activation may be one of the crucial, though underestimated, components of a deregulated host response to infection during SAKI.

## 1. Introduction

SAKI is a frequent complication in critically ill patients. It has been demonstrated that sepsis is found in about 40–50% of patients with AKI and is associated with extremely high morbidity and mortality in intensive care unit (ICU) patients [1,2]. Interestingly, the study published by Mehta et al. [3] suggested that AKI may increase the risk of sepsis, as 40% of critically ill patients develop sepsis after AKI of any origin. The discrepancies could arise from the fact that it usually is formidable to define the precise time of either sepsis or AKI beginning. Moreover, sepsis and its treatment expose the kidney to further injury because a prior history of AKI, even with complete recovery, still took the risk of developing chronic kidney disease [4].

For a long-time, renal hypoperfusion and associated ischemia, through extensive renal tubular epithelial cells’ (RTECs) death leading to acute tubular necrosis, has been identified as the primary lesion in SAKI [5]. Several animal models have shown that SAKI can develop despite the absence of histological or immunohistological changes in kidney tissue. In a placebo-controlled ovine model of SAKI induced by continuous intravenous (i.v.) infusion of *Escherichia coli*, renal histology showed patchy and focal changes with no evidence of macrophage infiltration, activation of apoptosis or increase in neutrophil gelatinase-associated lipocalin (NGAL), as a marker of tubular injury [6]. However, animals with SAKI had higher renal blood flow than controls, distinguishing the above model from human SAKI [7]. Furthermore, SAKI may develop in the absence of renal hypoperfusion and the presence of normal or increased global renal blood flow [8,9]. According to the given evidence, previous proposals highlighting systemic hypotension, renal vasoconstriction and ischemic kidney injury as the main pathophysiological mechanisms involved in SAKI have been changed.

Recently, sepsis was defined as a life-threatening organ dysfunction caused by a dysregulated host response to infection [10]. The host immune response in sepsis is a complex occurrence, which involves an excessive inflammatory response to infection at the initial phase leading to tissue damage, endothelial dysfunction and multiorgan injury [11]. On the other hand, a significant proportion of patients with sepsis manifest immunological deficiencies, which are associated with detrimental consequences [12,13]. Considering AKI as a common form of tissue damage in sepsis, in this review we discuss the role of innate immunity in sepsis-induced AKI with particular emphasis on the kynurenine pathway (KP) as a modulator of the activity of the innate immune system in SAKI.

## 2. Experimental Models of SAKI

Although sepsis is the most frequent cause of AKI in critically ill patients, its pathophysiological mechanisms are not comprehensively understood and there is a lack of preventive therapies available. Advancements in comprehending the pathomechanisms of SAKI have been restricted by particular technical and ethical problems [14]. The majority of the current knowledge of SAKI has been gathered from in vitro studies and animal models of sepsis–cecal ligation, puncture (CLP) and injection with lipopolysaccharide (LPS, endotoxin) [15].

### 2.1. Sepsis–Cecal Ligation, Puncture (CLP)

CLP, an exceptional technique for studying SAKI, is based on drilling the cecal intestinal wall with a needle, resulting in the gradual release of the bacterial flora into the peritoneal cavity, leading to peritonitis and sepsis. Severe sepsis is associated with multiorgan failure, finally evolving into septic shock. Gram-negative bacilli are the predominant pathological bacteria invading after CLP with the ability to release LPS, which induces a systemic inflammatory response via Toll-like receptor 4 (TLR4). Two hours after CLP, the plasma levels of tumor necrosis factor α (TNF-α), interferon-gamma (IFN-ɣ), and interleukin 12 (IL-12) increased, and plasma creatinine levels were significantly higher at 2, 6, and 24 h after CLP compared to control animals. Moreover, at 2 h after CLP, the production of superoxide in RTECs was increased, whereas the mitochondria of these cells were commonly scattered, swollen and rounded, and a certain amount of mitochondria manifested vacuolization. Kidney adenosine triphosphate (ATP) levels were significantly decreased at the time [16]. When performed correctly, CLP has been regarded as the “golden standard” animal model of sepsis, reproducing several features of bacterial peritonitis in humans [17,18]. However, CLP holds a few weak points—the severity of sepsis varies according to the number of punctures, the length of the cecum ligated, individual surgical skills, and distinctive anaesthetic agents. Additionally, the procedure might occur abscess formation, resulting in local rather than systemic CLP-induced inflammation [17,18].

### 2.2. Cecal Content Injection (CCI)

CCI, being a modified CLP-induced type of sepsis model, displays similar pathophysiology to CLP concerning cytokine changes and survival rate. Like CLP, CCI induces sepsis by contamination with an endogenous microbial source. Hence, the results obtained from the indicated model can be directly compared with previous CLP-generated results. The added benefits of the CCI model include the ability to control the severity of sepsis and greater consistency [19].

### 2.3. LPS-Induced Sepsis

Another experimental model widely used in the study of sepsis, though seldom used in the study of SAKI, is purified LPS injection, also called endotoxin-induced sepsis. The intraperitoneal or intravenous route of LPS administration leads to systemic activation of innate immunity [15]. The above mechanism is predominantly mediated by the interaction of the bacterial products with TLR-4, which is expressed on the surface of innate immunity cells (monocyte/macrophage, dendritic cells [DCs]) as well as on multiple other cells, for example, RTECs [20]. Activation of Toll-like receptors (TLRs) on the cells belonging to innate immunity initiates the expression of genes with a potential proinflammatory phenotype needed for DCs maturation or macrophage polarization to the M1 phenotype, which is essential for the induction of the pathogen-specific immune response. All TLRs, except TLR-3, utilize the myeloid differentiation factor 88 (MyD88)-dependent pathway, resulting in the activation of the nuclear factor kappa-light-chain-enhancer of activated B cells (NF-κB) and activator protein 1 (AP-1) [21]; in addition, animals with TLR-4 or MyD88 deficiency are unresponsive to LPS, pointing out that these adapter proteins are substantial for LPS-induced inflammatory response [22]. LPS-injected animals display biochemical and physiological changes resembling the fulminant forms of Gram-negative bacterial infection in humans, like systemic hypotension, lactic acidosis, impaired myocardial contractility, increased circulating levels of TNF-α, interleukin 6 (IL-6) and high-mobility group box 1 (HMGB1) [15]. The mitochondrial ultrastructure impairments, such as damaged cristae and impaired integrity of both internal and external mitochondrial membranes, have also been reported in LPS-induced SAKI in RTECs and the vascular endothelium [23].

LPS-induced sepsis varies from the CLP-induced model, because the stimulus given at once leads to a distinct kinetic release of inflammatory cytokines compared with the gradual release using the CLP technique [24]. The results obtained based on animal sepsis models shall be interpreted with great caution, while reactions to sepsis might vary essentially from humans. The activation of the innate immune system in animal models could potentially provide harmful effects, hence any sort of intervention attenuating the inflammatory response would be counterproductive. On the other hand, sepsis in humans is triggered by an infection in which immunological responses to microbial struggles may have parallel beneficial and harmful effects [25].

## 3. Role of Kidney Cells in SAKI

SAKI is predominately the consequence of a host’s dysregulated inflammatory response against invading pathogens and toxins [26], and may be responsible for organ dysfunction and meagre outcome. Numerous studies confirm that sepsis in patients frequently leads to multiple organ failures. The kidneys are particularly vulnerable, primarily to soluble factors like cytokines, damage-associated molecular patterns (DAMPs) and pathogen-associated molecular patterns (PAMPs), which are released into the circulation [27]. In addition to the usual approach to innate immunity, which accentuates cell-based mechanisms, the importance of receptors in septic AKI has been demonstrated by Gonçalves et al. [28]. The authors focused mainly on TLRs and nucleotide-binding oligomerization domain-like receptors (NLRs), explaining that both receptor families are involved in the recognition of inflammatory molecules, alarmins, and necrotic cells as well as the recognition of microbial PAMPs, which is necessary to generate a proper immune response.

During sepsis, PAMPs and DAMPs-associated molecular pathways are released intravascularly and bind to TLRs and NLRs that are present on the surface of immune cells, initiating a downstream cascade of signals that will result in the synthesis and release of proinflammatory cytokines. RTECs also have an ability to express TLRs, especially TLR2 and TLR4, and the exposition of these cells to DAMPs and PAMPs filtered through the glomerulus, or peritubular capillaries increased oxidative stress, mitochondrial injury and cell apoptosis [29,30,31]. Krüger et al. [32] demonstrated that TLR4 was constitutively expressed in human kidneys and that damaged tubules were positive for HMGB1, an endogenous TLR4 ligand. In vitro stimulation of human RTECs with HMGB1 confirmed the above-mentioned ligand can stimulate proinflammatory response through TLR4 and that the released proinflammatory mediators can contribute to further tubular cell damage. In line with observations, kidneys with a TLR4 loss-of-function allele contained fewer TNF-α and monocyte chemoattractant protein-1 (MCP-1) [32]. During sepsis, endotoxin was bonded to TLR4 on S1 proximal tubule cells, which henceforward resulted in oxidative stress in cells of the S2 segment [30].

Except for RTECs, TLR-2 and TLR-4 are constitutively expressed in Bowman’s capsular epithelium, glomerular and endothelial cells. Additionally, their expression has the ability to upregulate in the presence of INF-ɣ or TNF-α [33].

Yasuda et al. [34] showed that TLR-9, the receptor for unmethylated cytosine–phosphate–guanine dinucleotides present in bacterial DNA (CpG-DNA), plays an essential role in sepsis and SAKI. Under ordinary circumstances, TLR-9 is detected on DCs [35], however, Liu et al. [20] reported TLR-9 could be expressed even in tubular epithelial and glomerular cells 24 h after the induction of sepsis. The above observations suggest that not only may the bacterial DNA disseminate throughout the systemic circulation affecting the kidneys [36], but kidney cells may express TLR9, contributing to the development of TLR9-dependant signalling. Other studies also confirmed that the occurrence of sepsis caused the significant up-regulation of TLR9, and the activation of the TLR-9-dependent pathway led to SAKI and impaired survival rate of experimental animals. On the other hand, distinct methods used for reducing the activity of the TLR-9 pathway counteracted unfavourable effects, protecting kidney function and improving survival rate [16,20,34,37,38]. All of the above findings indicate that TLRs, especially TLR9-dependant signalling, can have a significant role in the pathogenesis of polymicrobial sepsis and may contribute to the activation of the immune response, resulting in tubule–interstitial injury.

Clinical studies demonstrated that plasma from patients with SAKI contains factors that induced functional alterations and apoptosis of glomerular, tubular epithelial cells and podocytes [39,40]. The analysis of Lerolle et al. [41] also showed the presence of RTECs apoptosis and leukocyte infiltration in the postmortem autopsy of patients with septic AKI. The recent examination by Cantaluppi et al. [42] demonstrated that hypoxic conditions, treatment with LPS, proinflammatory cy0t0okines (TNF-α, IFN-ɣ) or septic plasma, induced both mitochondrial and death receptor-mediated pathways of RTECs apoptosis, disrupted their oxidative metabolism and altered RTECs polarity. Mitochondrial DNA (mtDNA) of RTCEs, which is released from damaged and/or dead cells, could be identified as a “danger” signal and is able to activate the TLR9 pathway, contributing to the activation of neutrophils and the initiation of systemic inflammatory response syndrome (SIRS) [43].

Tsuji et al. [16] investigated the role of mtDNA in the TLR9-associated CLP-induced model of SAKI in mice. They observed that TLR9-knockout mice had higher mtDNA levels in the peritoneal cavity, higher leukocyte migration to the peritoneal cavity and lower bacteremia at 24 h after CLP than control animals. CLP-generated renal mitochondrial injury in the proximal tubules was reversed in the early phase in TLR9-knockoutmice. Wild-type mice (WT) and TLR9-knockout mice were injected i.v. by the authors with mitochondrial debris, which contained appreciable amounts of mtDNA to elucidate the effects of mtDNA on immune response and kidney injury. The above procedure caused an immune response similar to that of CLP, namely an increase in plasma IL-12 levels, splenic apoptosis and mitochondrial damage. All of the enumerated effects were diminished in the TLR9-knockout mice. The authors showed that endogenous mtDNA, which may not originate from a bacterial source, could be the primary TLR9-dependent DAMPs contributing to AKI during polymicrobial sepsis.

The production of proinflammatory cytokines TNF-α, IL-1β and IL-6 by injured RTECs may also initiate paracrine signalling, which may inform the neighbouring cells to decrease their death at the expense of function.

A primary mechanism of RTECs’ dysfunction in mitochondrial injury causes caspase release and cell apoptosis [44]. A critical function of the mitochondria is to provide energy (ATP) to the cell through the electron transport chain in a process called oxidative phosphorylation. Under normal circumstances, RTECs use β-oxidation of fatty acids as an energy source, and aerobic respiration is the primary mechanism of ATP production. During sepsis, mitochondria can alter metabolic processes to adapt to oxidative stress conditions through the conversion of pyruvate to lactate, which is a less efficient pathway to produce ATP [45]. The abundance of experimental studies indicated that the generation of mitochondria-l oxidative stress in septic AKI led to the ischemic injury of RTECs [16,46,47].

Considering that replication is one of the most energy-consuming processes in cells, RTECs can undergo cell cycle arrest to avoid cell death due to energy failure. Kashani et al. [48] found that two markers of cell cycle arrest—the tissue inhibitor of metalloproteinase-2 (TIMP-2) and insulin-like growth factor-binding protein 7 (IGFBP7)—are the best predictors of the development of SAKI, supporting the significance of this mechanism in human sepsis.

Another functional tubular cell alteration in the course of SAKI is the loss of cell polarity by the inflammatory microenvironment with the concomitant increase of delivery of sodium and chloride to the distal tubule and consequently glomerular filtration rate (GFR) impairment [42,49]. Tissue perfusion is critical for the proper functioning of any organ, whereas sepsis causes remodelling in microvascular blood flow distribution throughout the body, even in the absence of macrohemodynamic instability [50]. As GFR is determined by intraglomerular hydrostatic pressure, constriction of the renal afferent arteriole concurrently with dilation of the efferent arteriole was introduced as a mechanism explaining a fall in intraglomerular pressure, which led to a loss of GFR [51]. Moreover, intrarenal blood flow redistribution occurs during sepsis, driving blood flow away from the medulla [52]. The harmful effect of uneven blood distribution during sepsis, when the fragile balance of oxygen supply versus demand is wavering due to renal microvasculature dysfunction, can be a cause of acute tubular necrosis in the renal cortex and medulla [41] due to hypoxia and the overproduction of reactive oxygen species (ROS), reactive nitrogen species and cytokines. Cantaluppi et al. [42] documented that management of oxygen carriers–perfluorocarbons improved RTECs viability, reduced their apoptosis and metabolic disturbances and preserved RTECs’ polarity. Additionally, perfluorocarbons promoted CD133+ renal progenitor cell proliferation and differentiation towards a tubular-like phenotype, favouring tissue regeneration. The aforementioned findings confirm that high oxygen tension has a prominent role in the maintenance of RTECs viability and function under ischemic conditions.

## 4. Innate Immunity in SAKI

**Figure 1:**
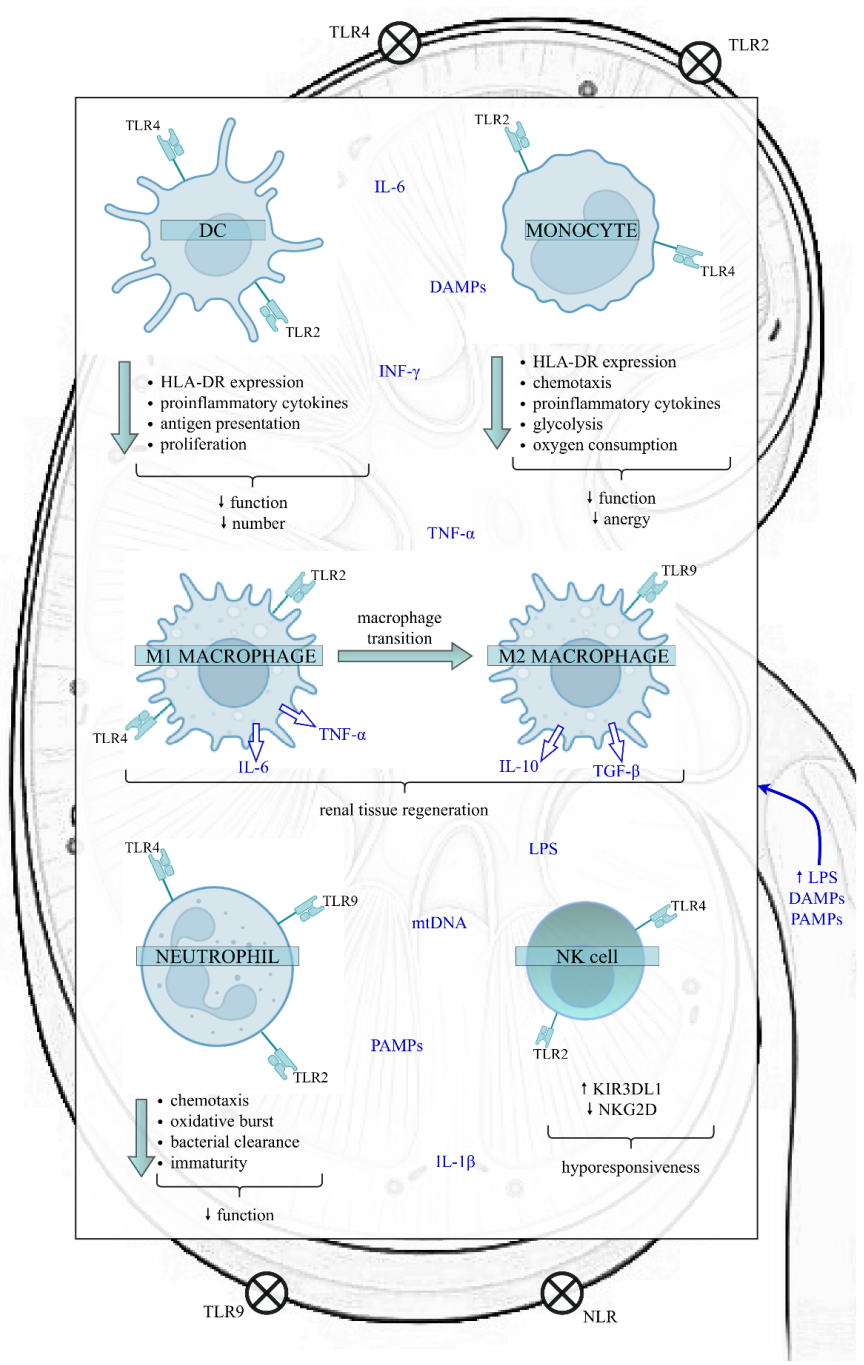
The impact of sepsis on the function of the innate immune cells in the kidney. Sepsis exposes the kidney to a proinflammatory microenvironment, which impairs various kidney structures, like RTECs, Bowman’s capsular epithelium, and glomerular and endothelial cells. In the initial phase of sepsis, LPS, PAMPs and DAMPs bind to TLRs and NLRs present on the surface of both kidney and innate immune cells, initiating a downstream cascade of signals that results in the synthesis and release of proinflammatory cytokines. However, as sepsis continues, the innate immune response is altered. The above alterations include the reduced function and number of DCs, diminished function and anergy of monocytes, the transition of M1 proinflammatory to M2 anti-inflammatory macrophages, increased proportion of immature neutrophils with a weakened role and a higher proportion of NK cells expressing the inhibitory receptor KIR3DL1. Nevertheless, data on the effects of sepsis on NK cells are insufficient. In contrast, the expression of activating membrane receptor NKG2D decreases, predisposing cells to hyporesponsiveness. Disturbances of innate immunity favour immunosuppression and can have harmful clinical consequences, such as increased susceptibility to secondary infections and viral reactivations, leading to increased mortality. Abbreviations: DC—dendritic cell; NK—natural killer; HLA-DR—human leukocyte antygen-DR; TLR—Toll-like receptor; NLR—NOD-like receptor; LPS—lipopolysaccharide; PAMPs—pathogen-associated molecular patterns; DAMPs—damage-associated molecular patterns; TNF-α—tumour necrosis factor alpha; IL-6—interleukin 6; IL-1β—interleukin 1 beta; IL-10—interleukin 10; TGF-β—transforming growth factor-beta; INF-ɣ—interferon gamma; mtDNA—mitochondrial DNA; KIR3DL1—Killer cell immunoglobulin-like receptor; NKG2D—NK group 2 member D receptor.

Sepsis can modulate the quantity and function of the most significant cells belonging to the innate immune system, such as dendritic cells (DCs), monocytes/macrophages, neutrophils and natural killer (NK) cells (Figure 1).

### 4.1. DCs in SAKI

DCs, as an essential component in the defence against invading pathogens, play a pivotal role in SAKI. Upon maturation and upregulation of MHC and other co-stimulatory molecules, they have the capacity to release multiple mediators, which modify the local environment affecting the activity of other immune cells. In the mouse model of systemic bacterial infection, Yatim et al. [53] presented that renal resident DCs can uptake intravascular pathogens and present them to effector T cells, mediating T cells’ migration into the kidney.

Generally, the quantity of DCs and their function were reduced by sepsis-induced apoptosis [54,55]. The loss of DCs was more extensive in patients with sepsis who died than in those who survived. It has been shown that LPS can markedly inhibit DCs proliferation in vitro [56]. The recovery to regular DC amounts and function lasted up to several weeks after sepsis and was presumably caused by epigenetic alterations [55,57], as epigenetic regulation at the promoter region of the IL-12 gene correlated with decreased IL-12 production in response to TLRs in lung-resident DCs from mice after sepsis [58]. The consequence of the impaired function of DCs after sepsis is an inability to present the proper antigen-dependent response, which leads to inappropriate naïve T-cell differentiation. DCs in sepsis exhibited decreased expression of human leukocyte antigen-DR (HLA-DR) and reduced secretion of proinflammatory cytokines upon stimulation by bacterial prducts [59]. The treatment of DCs with their growth factor—FMS-related tyrosine kinase 3 ligand (FLT3L) restored DCs’ numbers and function [54]. Moreover, blocking the sepsis-induced overexpression of CD155 on DCs, which is known as an inhibitor of T cells, protected mice from sepsis-induced mortality [60]. Another study showed that sepsis-induced cellular and cytokine microenvironments induced a shift in DCs towards a tolerogenic profile, driven by the production of transforming growth factor β (TGF-β) and induction of regulatory T cells (Tregs). The indicated phenomenon predisposed animals to secondary infections in the long term [61], confirming the fundamental role of DCs in sepsis-induced immunosuppression [62]. DCs possess various innate receptors, including TLRs, which upon activation, initiate an inflammatory response. TLR9 is primarily expressed in DCs, and to a lesser extent, macrophages [38,63]. Deletion of the receptor attenuates SAKI [34], and the adoptive transfer of TLR9-deficient DCs has been demonstrated as a factor in increasing survival in sepsis [38]. Tsuji et al. [16] have determined that circulating mtDNA, endogenously released during sepsis, is a major upstream factor of the TLR9 pathway. The same group of researchers, using the CLP model, discovered that DCs activated by the TLR9/IL-17A axis have an ability to secrete IL-23, which leads to stimulation of IL-17A production in T cells during sepsis, contributing to the development of SAKI [64].

The triggering of TLR4 on DCs by LPS causes spleen tyrosine kinase (Syk) activation and the production of proinflammatory cytokines, including IL-6 and MCP-1 [65], which participate in the development of SAKI [28]. Al-Harbi et al. [66] showed that blockade of the Syk/IL-6/MCP-1 pathway in DCs during LPS-induced AKI repairs of tubular structures in the kidneys attenuates SAKI. These results documented that the inhibition of Syk signalling in DCs might present an effective strategy to limit inflammatory cascade during AKI.

The binding of LPS to TLR4 on the surface of DCs may activate nuclear factor NF-κB [67], which controls the expression of many proinflammatory cytokines [68]. In the LPS-induced model of SAKI, it has been demonstrated that the suppression of abnormal immune responses through inhibition of the TLR4/NF-κB pathway might prevent AKI and improve the clinical outcome of sepsis. The in vitro experiment performed in the study also confirmed that LPS stimulated DCs by TLR4/NF-κB signalling, and that inhibition of the TLR4/NF-κB pathway in DCs is associated with reduced production of proinflammatory cytokines by the indicated cells [56].

Bruton’s tyrosine kinase (BTK) is involved in inflammatory and oxidative signalling in diverse immune cells, including DCs [69]. BTK regulates signalling downstream of the TLRs, which includes phospholipase C, nuclear factor of activated T cells (NFAT), NF-κB, and MAP kinases, leading to cellular activation, cytokine release, and oxidant generation [70]. Nadeem et al. [71] showed that the ratio of BTK-positive CD11c+DCs increased during LPS-induced SAKI, which was associated with concomitant induction of oxidative stress in these cells. As oxidative stress contributes to the activation and amplification of the inflammatory potential in DCs [72], they suggested that activation of TLR4 upregulates BTK signalling in DCs, which is likely an essential contributor to the development of sepsis-induced AKI. Then, they used an inhibitor of BTK—ibrutinib, and confirmed that BTK inhibition could be responsible for the improvement of AKI through the attenuation of oxidative stress in innate immune cells [71].

### 4.2. Monocytes/Macrophages in SAKI

Among innate immune cells, monocytes and macrophages are essential for the initiation of sepsis, as they release various proinflammatory cytokines and chemokines, TNFα, IL-6, MCP-1 and intercellular adhesion molecule 1 (ICAM-1), which can cause kidney damage and SAKI, resulting in a high mortality rate. During sepsis, major metabolic and epigenetic reprogramming occurs, which leads to pivotal shifts in transcriptional profile, resulting in the induction and maintenance of sepsis-induced immunosuppression [73].

Several metabolic defects have been described in patients with sepsis, for example, decreased glycolysis and oxygen consumption in monocytes or a dysfunctional mammalian target of the rapamycin (mTOR) pathway [74]. Weisheit et al. [75] observed differential methylation of CpG sites related to metabolic activity in human peripheral blood mononuclear cells (PBMCs) 18 h after septic challenge.

The previously mentioned alterations in metabolic processes were associated with changes in the inflammatory capacity of monocytes;the expression of hypoxia-induced genes was upregulated in monocytes from septic patients, increasing IL-1 receptor-associated kinase M (IRAKM), which reduces the expression of proinflammatory genes, promoting immunotolerance [76]. The reduction in HLA-DR expression [77] or programmed death-ligand 1 (PDL1) overexpression by monocytes [78] has been described as the essential feature of monocyte anergy. Both of them were considered independent predictors of mortality after sepsis [77,78]. Moreover, the distinct cytokine production profiles in human monocytes in hyperinflammatory circumstances and the immunotolerant state after sepsis are also associated with alterations in cellular metabolism. The decreased capacity of monocytes collected from patients with sepsis to release proinflammatory cytokines in response to LPS, TLR agonists, or various bacterial compounds can lead to sepsis-induced immunotolerance. Similarly, prestimulation of monocytes with high doses of LPS can induce immunoparalysis [75]. Therefore, it seems that a cytokine storm at the beginning of sepsis may cause the loss of specific epigenetic marks at promoters of proinflammatory genes in monocytes or macrophages, promoting their tolerogenic state.

In the SAKI model in mice, Zhang and coworkers observed that 24 h after intraperitoneal LPS injection, peripheral blood monocytes showed only subtle change, but nevertheless, their population increased significantly at 48 h. Within a few hours after LPS injection, rapid infiltration of neutrophils and macrophages was observed in the kidney of animals, which was accompanied by the production of proinflammatory factors, the generation of oxidative stress, apoptosis and kidney function impairment. The pretreatment of LPS-injected mice with extracts of *Hydrangea paniculata*, a traditional Chinese medicinal plant with antioxidant and anti-inflammatory properties, was able to reduce the unfavourable LPS-dependent effects, protecting kidneys from SAKI [79]. In line with the aforementioned observations, histological data show that kidneys from septic patients, compared with nonseptic controls, have increased infiltration of monocytes in the glomeruli and interstitial capillaries and a higher percentage of tubular cell apoptosis [41,80].

Renal mononuclear phagocytic cells include diverse subtypes, and the dual role of the above cells in processes of damage and repair during SAKI has been observed. It has been shown that type I (M1) macrophages are involved in inflammatory tissue destruction, while type II (M2) macrophages are active during anti-inflammatory response and tissue repair [81,82,83,84,85]. Aslan et al. [80] observed an accumulation of M1 and M2 types of macrophages around the glomeruli and in the glomerular capillaries, though not in the tubulointerstitium of patients with SAKI. However, M2 macrophages were predominantly shown in the glomeruli of patients with SAKI compared to controls, and in some patients with SAKI, markers characteristic of both type M1 and M2 macrophages were detected. Interestingly, there were significantly more proliferating cells in both the glomeruli and the tubulointerstitium, whereas apoptotic cells were nearly absent in the glomeruli of patients with SAKI compared to controls. Although the identity of proliferating cells was not explained by the authors, their presence indicates increased regeneration of renal tissue as a mechanism of repair. The above results indicate that the process of inflammation and repair in SAKI can occur simultaneously in the same area of kidney tissue. Even though M2 macrophages present in SAKI glomeruli may originate from systemic circulation, the transition from M1 to M2 observed in the study suggests that M2 macrophages may arise locally from activated, proliferating cells. The present observation was in line with previous experiments in animal models [86,87]. The recent study by Yao et al. [88] in CLP-induced SAKI also demonstrated that during the early stage of sepsis, M1 macrophage infiltration and production of proinflammatory cytokines–IL-6 and TNF-α was observed in renal tissues and was inhibited by the application of the macrophage infiltration inhibitor heparin. A similar effect was observed in human SAKI, in which the removal of macrophage migration inhibitory factor (MIF) from the plasma through dialysis was accompanied by improved survival [89]. The usage of hydrogen, an effective anti-inflammatory antioxidant in CLP-induced SAKI, resulted in the expression of anti-inflammatory cytokines IL-4 and IL-13 in renal tissues and promoted M2 macrophage polarization, with the generation of additional anti-inflammatory cytokines: IL-10 and TGF-β. Subsequently, in LPS-stimulated RAW 264.7 cells, it has been confirmed that hydrogen promoted M2 generation, as well as IL-10 and TGF-β production. Moreover, the authors noticed that the mechanism by which hydrogen might stimulate its anti-inflammatory effects was, to an extent, dependent on the TGF-β pathway, as the inhibition of the TGF-β1 receptor partly reversed the consequences of the hydrogen influence on kidney function and survival rate in septic animals [88].

The macrophage phenotype might be represented by the expression or repression of iron-regulated genes. The proinflammatory M1 macrophages sequester iron as part of the host defence against pathogens, whereas the anti-inflammatory M2 phenotype increases its phagocytic activity and releases iron to the microenvironment, elevating cell proliferation, tissue repair and regeneration [90,91]. Recently, Mertens et al. [92] found that at 48 h after the CLP procedure, iron-released macrophages also produce and secrete iron-loaded lipocalin-2 (LCN-2), which was associated with renal regeneration markers. The same group of scientists previously demonstrated that LCN-2 was able to perform as part of a complex network of iron-regulated genes in order to promote cellular proliferation [93].

### 4.3. Neutrophils in SAKI

Neutrophils are released into the bloodstream from the bone marrow, where they circulate as the first responders of the innate immune system against acute injury or invading pathogens. They are remarkably mobile and have the ability to quickly reach the site of inflammation, where they destroy the pathogen by phagocytosis or degranulation. Neutrophils also release neutrophil extracellular traps (NETs), which can trap and neutralize pathogens [94]. Several consecutive responses occur between the circulating neutrophils and the vascular endothelium, such as capture, rolling, adhesion, and transmigration, allowing them to enter damaged tissue [95,96].

Neutrophils are prominent participants in SAKI, their infiltration is found in the renal biopsies from SAKI patients and murine SAKI models [41,71,80]. Neutrophils infiltrate renal tissue under chemotactic signals generated by chemokines and cytokines, including C-X-C motif chemokine ligand 1 (CXCL1), MCP-1, and interleukin-17A (IL-17A), which are released by activated macrophages, DCs and T cells during AKI. Studies investigating the molecular mechanisms of neutrophils’ recruitment into the kidney discovered that two β2-integrins, macrophage-1 antigen (Mac-1) and lymphocyte function-associated antigen 1 (LFA-1); two adhesion molecules, ICAM-1 and vascular cell adhesion molecule 1 (VCAM-1); as well as E-selectin and P-selectin are involved in neutrophil recruitment into the kidney after the induction of sepsis. The elimination of neutrophils or the blocking of one of the previously mentioned molecules reduces the number of adherent neutrophils and abolishes SAKI in a CLP-induced sepsis model [97,98]. In experimental sepsis, the activation of neutrophils resulted in down-regulation of the expression of their chemokine receptor C-X-C Motif Chemokine Receptor 2 (CXCR2), which impaired the migration of cells to the site of infection, while up-regulation of the receptor expression mediated infiltration of neutrophils in the lungs, kidneys and heart. The above-mentioned changes in the expression of neutrophil chemokine receptors have been likewise observed in septic patients [99].

The migration of neutrophils to the kidney during sepsis is dependent on the activation of TLR2, TLR4 and the adapter molecule-MyD88. The genetical depletion of TLR2, TLR4, and especially MyD88 in CLP-induced sepsis resulted in reduced neutrophils infiltration into the kidney, decreased expression of proinflammatory cytokines, iNOS, MPO and a significant improvement in kidney function, indicating that neutrophils participate in polymicrobial SAKI mainly through the MyD88 pathway [100].

Neutrophils possess enzymes—NADPH oxidase (NOX-2), inducible nitric oxide synthase (iNOS) and myeloperoxidase (MPO)—which produce the oxidants superoxide and nitric oxide. The indicated oxidants have the ability to transform into other oxidizing species, such as hydroxyl radical, hydrogen peroxide, hypochlorous acid and peroxynitrite. All of the compounds are required to be scavenged by the antioxidants, otherwise, oxidation of lipids, proteins, and DNA occurs, causing cellular dysfunction [101]. The adherent and transmigrated neutrophils, through the activation of the NOX2/iNOS/MPO system, release ROS, myeloperoxidase and other enzymes that have the potential to damage glomerulus and renal tubules [102,103].

In general, profound alterations in the characteristics and functions of neutrophils were described in patients during the first week after sepsis, including decreased chemotaxis, reduced oxidative burst and impaired bacterial clearance [104]. Moreover, an increase in the percentage of immature neutrophils with immunosuppressive functions was observed, which was associated with increased early mortality after sepsis [104,105]. The decreased response of neutrophils to N-formyl-methionyl-leucyl-phenylalanine (fMLP) stimulation, reflecting the responsiveness of the circulating neutrophils for bacterial-derived activating agonists, was observed in patients with trauma, who subsequently developed septic shock [106]. Similarly, in a mouse model of sepsis, accumulation of HMGB1 in the late phase of sepsis contributes to neutrophil defects and may be responsible for increased susceptibility to secondary infections [107]. Furthermore, in both patients and animals with sepsis, neutrophils can overexpress PDL1, which is associated with harmful outcomes [108].

Non-receptor protein tyrosine kinases, such as IL-2 inducible T-cell kinase (ITK) or BTK, play crucial roles in the downstream signalling of several cell surface receptors in immune and non-immune cells. In LPS-induced SAKI in mice, blocking both ITK and BTK by specific inhibitors, caused diminished neutrophil infiltration, reduced MPO activity and oxidative damage in renal tissue during AKI, which was associated with the restoration of biochemical parameters of kidney function [71,109].

Syk is another type of kinase sited in neutrophils, playing an essential role in surface receptor signaling, and the triggering of TLRs on cells by LPS causes Syk activation and ROS production. Blockade of Syk signalling in LPS-induced AKI led to the improvement of SAKI as depicted by the decrease of iNOS, NOX2 and nitrotyrosine in neutrophils, reduced renal MPO activity, repair of tubular structures in the kidneys and the enhancement of biochemical markers of kidney function [66].

Based on the above experimental research, it can be concluded that neutrophil recruitment into the kidney, especially in later stages of SAKI, may contribute to the injury of glomerulus and renal tubules resulting in hemodynamic disturbance and tubular dysfunction. Speculatively, the regulation of the neutrophils’ function and their uncontrolled infiltration into renal tissue could present an effective therapy to protect against SAKI.

However, data from human studies provided inconsistent results. Histological data on post-mortem biopsies taken immediately after patients’ deaths caused by SAKI showed only limited inflammation, which involved predominantly mononuclear capillary leukocytic infiltration [41,110]. Instead, Aslan et al. [80] observed notable neutrophil infiltration in the glomeruli, tubulointerstitium, and the peritubular capillaries in SAKI compared to controls, albeit they claimed that no renal cell injury could be attributed to the neutrophil influx. In the following study [111], neutrophil activation markers activin A (a cytosolic neutrophil protein), interleukin 8 (IL-8) (a chemoattractant for neutrophils) and MPO (a neutrophil biomarker released in tissues) were determined in plasma and urine in critically ill sepsis patients with AKI. All the markers were increased in the plasma of AKI patients 24 h after admission to the ICU compared to non-AKI subjects. There were correlations between the plasma and urine concentrations of activin A and IL-8. However, plasma MPO did not correlate with urinary MPO, indicating that urine MPO reflects the MPO released from the renal parenchyma. Urine MPO was related to urine IL-8 level and the presence of SAKI, suggesting that renal accumulation of neutrophils may be associated with SAKI. Further, the same group of researchers confirmed that during sepsis, neutrophil activation could be detected hours before the emergence of organ dysfunction, such as AKI [112].

In conclusion, the function of neutrophils in SAKI seems ambiguous, as, on the one hand, they are involved in the formation of inflammation-causing destruction of surrounding tissues. On the other hand, they are necessary for tissue regeneration in human SAKI [113]. Accordingly, the exact function of neutrophils in human SAKI requires further research.

### 4.4. NK Cells in SAKI

NK cells are involved in the early response to bacterial infections and may also affect the adaptive immune response through direct interaction with DCs or the production of cytokines. The function of NK cells is the result of interaction between activating receptors, which detect injured cells displaying altered class I MHC, and inhibitory receptors, which identify accurate cells by recognition of their unimpaired class I MHC expression [114]. Activated NK cells might exert cytotoxic activity against tumour cells expressing MHC class I molecules [115], proving that attacking overactivated ordinary cells is possible. While NK cells are activated by cytokines, bacterial compounds or LPS, they exert cytotoxic activity by releasing cytotoxic mediators perforin and granzyme, or by producing proinflammatory cytokines including IFN-γ, depending on the type of stimuli [114].

The murine models of sepsis have shown a detrimental proinflammatory effect on NK cells, which were a principal source of IFN-γ. Early depletion of NK cells led the improvement in the survival of sepsis-challenged mice [116,117,118,119]. Although mouse NK cells may be involved in some renal diseases, including AKI [120,121], there is no data on the role of these cells in the course of SAKI.

Human NK cells are defined as CD56+ CD3- (CD56+ NK cells) large granular lymphocytes [122]. The first study to provide an extensive assessment of circulating NK cells in critically ill septic patients early (<48 h) after admission to the ICU was conducted by Forel et al. [122]. They showed that the proportion of CD56+ NK cells was similar in sepsis patients and healthy controls, whereas the total number of CD56+ NK cells was reduced in the sepsis group. In contradiction to murine data, they did not observe an overactivated status in septic patients, as was demonstrated by the lack of difference in the NK-cell cytotoxicity test or even reduced INF-γ production by the NK cells of septic patients compared to controls. Moreover, they did not observe any correlation between the amount and function of circulating NK cells upon ICU admission and mortality, even in septic patients. The only significant difference observed in the above study was a higher proportion of NK cells expressing the inhibitory receptor KIR3DL1 in patients with sepsis compared to those who were non-septic. The results suggest that in critically ill patients with sepsis, NK cells show hyporeactivity that may be part of immunotolerance or immune paralysis [122]. The above hypothesis stood in agreement with a previously made observation that the expression of activating membrane receptor NKG2D in NK cells decreased in patients receiving dialysotherapy [123], which could be associated with impaired immune functions and increased frequency of infections in these patients.

Healthy kidneys contain a considerable number of CD56+ NK cells. CD56 bright NK cells’ phenotype is identified as resident cells in renal tissue, which may play a crucial role in defence against infections. On the other hand, the stimulation of cells by a combination of IL-2, IL-12 and IL-15 can cause potent cytotoxicity against intrinsic renal cells, leading to renal tissue damage [120]. While studies considering renal NK cells are limited, it was suggested that CD56 bright NK cells in the kidney may cause pathogenic effects, referencing their increased number in patients with IgA nephropathy [124] or fibrosis [125]. Due to the lack of studies on the role of CD56+ NK cells in SAKI, further investigations in the above-mentioned field are necessary for the future.

## 5. Kynurenine Pathway (KP) Activation in SAKI

Tryptophan (TRP) catabolism by KP is a major metabolic pathway utilized by several cells of innate immunity to maintain immunological tolerance. The catabolic pathway of TRP commences with the activation of enzyme-indoleamine 2,3-dioxygenase 1 (IDO1) [126], which is highly expressed in antigen-presenting cells (APCs) such as DCs, macrophages and multiple other cells belonging to the innate immune system. In addition to being involved in the innate immune response trough IDO1, the kynurenine pathway is also the main catabolic route for TRP metabolism through tryptophan 2,3-dioxygenase 2 (TDO2) [127,128,129,130,131].

The activation of IDO1 has been considered an immune checkpoint mechanism capable of promoting immunotolerance. The activation of IDO1 in the cells of innate immunity depletes TRP from the microenvironment, restricting the proliferation and proper function of T cells. On the other hand, TRP metabolites—kynurenines, which are ligands for aryl hydrocarbon receptor (AhR), can induce immunotolerance through additional mechanisms involving the development and expansion of regulatory T cells (Tregs) and T helper 2 (Th2), with simultaneous extenuation of inflammatory Th1 and Th17-mediated responses. For an in-depth review of the role of IDO1 and KP activation in immunotolerance, the reader is referred to Krupa and Kowalska [132].

In the current literature, only two studies concerning KP metabolites in SAKI were published [133,134]. Dabrowski et al. [133] analysed plasma kynurenic acid (KYNA) concentration in septic shock patients with AKI undergoing continuous veno-venous haemofiltration (CVVH). Measurements were conducted a few times: before CVVH and at various time points after the beginning of the CVVH procedure. The authors showed that an unsuccessful reduction of KYNA after CVVH treatment might predict fatal outcomes. However, the pathophysiology and reasons for insufficient KYNA clearance in non-survivors were not addressed.

Using an experimental model of polymicrobial sepsis (CLP), Iwaki et al. [134] demonstrated that mice deficient in peroxisome proliferator-activated receptor α (PPAR-α) expression had impaired kidney function with the alterations presented mainly in the proximal tubules. The expression of markers of kidney injury and inflammation were elevated in the renal tissue of PPAR-α knockout mice compared with the controls. The metabolomic analysis revealed metabolic disturbances in several pathways, including the TRP/KYN/KYNA/NAD+ system in PPAR-α knockout mice after CLP induction. Septic PPAR-α knockout mice had lower levels of TRP and niacinamide—metabolites essential to NAD+ biosynthesis—as well as lower concentrations of shikimic acid, a precursor of TRP biosynthesis related to NAD+ metabolism. About 95% of the body’s TRP is metabolized to NAD+ [135], and PPAR-α regulates the activity of enzymes in the TRP/KYN/NAD+ biosynthetic pathway, including quinoline phosphoribosyltransferase [136]. The authors suggested that PPAR-α-induced disturbances of the kynurenine pathway may play a substantial role in providing cellular energy in the course of sepsis. Provided experimental data were verified in children with sepsis whose genome profiles were characterized by repression of the PPAR-α signalling pathway. Corroborative results indicated that similar PPAR-α-dependent mechanisms might also exist in human SAKI.

## 6. IDO1, KP Activation and Innate Immunity in Sepsis

So far, the role of IDO1 and the KP metabolites in sepsis and septic shock remains unresolved; therefore, in the below section of our review, we focused on exhibiting the available data on the topic. The role of the inflammatory response during sepsis should be to restrict the area of damage, stop the infection from spreading, and repair damaged tissue afterwards. Activation of IDO1 by proinflammatory cytokines and degradation of TRP by IDO1-expressing cells of the innate immune system (DCs, monocytes/macrophages and neutrophils) is considered the elementary mechanism of defence against infection, since numerous microbial organisms need that essential amino acid for replication [137]. Moreover, TRP metabolites, such as KYN or 3-hydroxykynurenine (3-HKYN), possess an antibacterial effect, directly suppressing pathogen replication [138]. The recognition of LPS by TLRs on the surface of APCs initiates intracellular signal transduction, NF-κB activation and transcription of genes encoding proinflammatory cytokines. Among APCs, DCs are considered the most potent, because they are the most proficient APCs for inducing T-cell-mediated immunity against diverse antigens; consequently, they may perform as a link between innate and adaptive immune responses [139].

### 6.1. Data on Human Studies

The clinical studies clearly show that IDO1 and the activation of KP are associated with the course of sepsis and mortality prognosis. Plasma IDO1 activity gradually increased according to sepsis severity, and septic patients who died had higher IDO1 activity on admission than individuals who survived. The percentage and the absolute number of circulating monocytes increased in septic patients, and they were a primary source of active IDO1 in peripheral blood. Moreover, the circulating monocytes collected from septic patients were not able to activate IDO1 through TLRs engagement, while they remained responsive to IFN-ɣ stimulation [140]. Similarly, Huttunen et al. [141] revealed that IDO1 activity was remarkedly increased in patients with bacteriemia. Therefore, increased IDO1 activity remained an independent risk factor for severe disease and case fatality. Schefold et al. [142] demonstrated that IDO1 activity and metabolites downstream of IDO1, such as KYN, KYNA and quinolinic acid (QUIN), were increased in the septic patients’ blood. Subsequent, they used stimulation with granulocyte–macrophage colony-stimulating factor (GM-CSF), which has been shown to reverse monocytic deactivation in patients with sepsis [143]. They observed the reduction of IDO1 activity and levels of KP metabolites in septic patients receiving GM-CSF therapy. The decline in elevated IDO1 activity and kynurenines were associated with normalization of monocytic function, assessed by monocytic HLA-DR expression and TNF-α release, as well as with diminished serum procalcitonin level, reflecting the degree of the bacterial load. The aforementioned results showed that IDO1 activity and TRP metabolism in sepsis might translate into impaired monocyte function and sepsis-induced immune dysfunction, which usually would be observed in the later stage of the disease.

### 6.2. Data on Experimental Sepsis Model

IDO1 expression and function in cells of innate immunity are dependent on the type of immune cells and the origin of the stimulus. It has been reported that IDO1 is an essential enzyme whose activity produces the pro-inflammatory cytokine IL-12 in DCs [144]. The activity of IDO1 in DCs has a significant role in the survival from LPS-induced septic shock [145]. Yim et al. [19] administered 1-methyl-D-tryptophan (1-MT), a competitive inhibitor of IDO1, before inducing sepsis in mice and observed an increase in the survival rate and tendency to upregulate serum IL-10/IL-12 ratio at 48 h after CCI. The effectiveness of 1-MT was further confirmed by the adoptive transfer of bone marrow-derived DCs, cultured in vitro with LPS and 1-MT, which also increased the survival rate in the model.

Similarly, the pharmacological blockade or genetic deletion of IDO1 in endotoxin-administered mice caused a decrease in the levels of proinflammatory cytokines TNF-α, IL-6, and IL-12 with simultaneous enhancement of IL-10 concentration. The altered balance between pro- and anti-inflammatory cytokines improved survival in murine endotoxemia 48 h after the LPS dispensation [145]. In the study, IDO1 expression was increased by exogenous IL-12 while being decreased by exogenous IL-10 in DCs and splenic DCs. However, the net effect on IDO1 expression in DCs was dependent on the balance of IL-12 and IL-10; meanwhile, the balance became reversed by blocking IDO1. The above findings indicated that endotoxin-induced IDO1 could trigger a proinflammatory response in DCs, which might potentially become detrimental in sepsis.

Hoshi et al. [146] examined the role of IDO1 in bacterial peritonitis and sepsis in the CLP model using 1-MT, IDO1-knockout mice and chimeric mice, in which the bone marrow-derived cells were IDO1 deficient. After the CLP procedure, IDO1 expression in the peritoneal CD11b(+) cells and serum KYN levels increased. Both 1-MT treatment and IDO1 deficiency, particularly in chimeric animals, reduced mortality after CLP. IDO1-knockout mice showed increased recruitment of neutrophils and mononuclear cells into the peritoneal cavity, decreased bacteriemia in the blood, and higher expression of chemoattractant chemokine CXCL-1 and CXCL-2 in the peritoneal cells compared to controls. Moreover, IDO1 induction with LPS inhibited chemokines production in cultured peritoneal cells stimulated with LPS. Based on the obtained results, it was established that IDO1 increased mortality rates through a decrease in recruited neutrophils and mononuclear cells into the infection focus via a reduction in chemokine production. Thus, blockade of IDO1 in the model seemed to play a beneficial role in host protection during bacterial peritonitis and sepsis.

Although the LPS-mediated activation of proinflammatory cytokines is fundamental for adequate host defence, an out-of-control immune response provokes profound tissue damage, leading to septic shock. IDO1, as a TRP-catabolizing enzyme, is able to deplete intracellular or microenvironmental TRP concentrations, detected by the general control nonderepressible 2 kinases (GCN2K) [147]. The activation of GCN2K regulates the expression of several proinflammatory cytokines, such as IL-6 and IL-12. Liu et al. [148] demonstrated that activated macrophages could express IDO1, and that depletion of their own TRP supply serves as a crucial second signal activating the GCN2K pathway to augment proinflammatory cytokine production and mortality in a mouse model of septicemia. GCN2K knockout macrophages exhibited a significant reduction in cytokine expression after LPS stimulation, and GCN2K knockout mice had reduced inflammatory responses, which correlated with reduced mortality. Consequently, IDO1-dependent TRP depletion in macrophages could synergize with proinflammatory signals generated by endotoxemia to potentially reinforce innate immune responsiveness with a conceivable unfavourable prognosis.

### 6.3. IDO1, KP Activation and Sepsis-Induced Immunosuppression

Behind the hyperinflammatory immune response in endotoxin-induced sepsis, a compensatory hypoinflammatory phase follows, protecting the organism against secondary, extensive secretion of proinflammatory cytokines. The mechanism acts as a negative feedback loop to restrain the inflammatory response and is involved in endotoxin tolerance up to complete paralysis of the immune system [149].

However, it remains unclear whether the increased IDO1 activity contributes to excessive inflammation or reflects a compensatory response to an inflammatory state, enabling inducing immune tolerance.

In animal models of sepsis, pharmacological or genetic depletion of IDO1 before LPS admission was associated with protection against LPS-induced septic shock at 24 or 48 h after sepsis induction [19,145,146]. In human studies, a high KYN/TRP ratio, an indicator of the activation of first-line innate immune defence, has been used as a marker of poor outcome and mortality in critically ill patients with sepsis [141,150]. However, several clinical studies focused only on tempering the hyperinflammatory initial phase and failed to decrease 30-day mortality in patients with sepsis [151,152]. The failure of the anti-inflammatory therapies in these conditions might be partially explained by the fact that patients may exhibit both hyperinflammation and immunosuppression concomitantly, with one being transiently dominant over the other [149].

In mammals, the first step in TRP catabolism can be performed independently by IDO1, its paralogue–IDO2 and TDO2—predominantly expressed in the liver [153]. Bessede et al. [154] studied the role of AhR and TRP catabolism in primary LPS responsiveness and the induction of endotoxin tolerance using AhR-deficient mice and animals lacking IDO1, IDO2 or TDO2. They found that an overly inflammatory response to direct LPS challenge was relieved by AhR and TDO2-dependent TRP metabolism to downregulate early inflammatory gene expression, whereas the generation of endotoxin immunotolerance after LPS rechallenge mainly occurred in DCs and required the combined effects of AhR, IDO1 and TGF-β. Interestingly, supplementation with KYN after 6 h of LPS rechallenge was not able to restore tolerance in the absence of IDO1, which was associated with a marked reduction in TGF-β production in animals, suggesting that IDO1 was essential for circulating TGF-β production. The authors also demonstrated that AhR-associated tyrosine kinase Src activity is responsible for IDO1 phosphorylation and TGF-β production by IDO1-competent DCs. Moreover, AhR was responsible for protection against *Listeria monocytogenes* by decreasing activation of the nuclear factor kappa-light-chain-enhancer of activated B cells (NF-κB) and reducing the expression of pro-inflammatory cytokines (IL-6 and TNF-α) in macrophages [155].

Yamamoto et al. [156] investigated the role of IDO2 in innate immunity using LPS-induced sepsis in IDO2-knockout mice. They noticed that IDO2-knockout animals had higher mortality, increased production of proinflammatory cytokines in serum, and higher signal transduction and activation of transcription 3 (STAT3) phosphorylation in the spleen than WT mice. Moreover, the peritoneal macrophages from IDO2-knockout mice stimulated with LPS produced more cytokines than controls. In the murine macrophage cell line overexpressing IDO2, Yamamoto and colleagues demonstrated that the overexpression of IDO2 did not alter NF-κB or STAT1 expression though decreased IL-6 and STAT3 expression in these cells, proving that IDO2 contributes to cytokine production and degradation through IL-6/STAT3 signalling without changes in the KYN concentration. Previously, IDO2 induction was observed in the other cells of the innate immune system, such as DCs and mesenchymal stem cells, in response to IFN-ɣ [157]. The indicated data suggest that IDO2 is an essential negative regulator of cytokine signalling and the maintenance of cytokine homeostasis in the innate immune system.

KYN and its metabolite KYNA, by binding to AhR, resulted in the production of anti-inflammatory cytokines, such as IL-10 and TGF-𝛽, and the KYN/AhR axis was involved in the generation of Tregs and the creation of an anti-inflammatory microenvironment [158,159]. It has been shown that the increase of Tregs is deleterious in sepsis patients and associated with decreased proliferation of effector T cells [160]. A similar observation accomplished by Darcy et al. [161], who demonstrated that elevated KYN/TRP ratio in patients with sepsis coexisted with reduced CD4+ and CD8+ T-cell counts, pointing to an overall impairment of immune functions. Meanwhile, AhR-deficient mice were more sensitive to endotoxin shock than WT mice, indicating the role of AhR in modulating the TLR-4-induced inflammatory response [162]. Moreover, KYN activated the AhR-mediated transcription of IL-6, and in trauma patients with increased IDO1 activity, the nonsurvivors had higher concentrations of IL-6 after trauma than survivors [150].

The intriguing results were presented by Wirthgen et al. [163], who investigated the protective and immunomodulatory effects of 1-MT against endotoxin-induced shock in a porcine in vivo model. They showed that pretreatment with 1-MT increased TRP and its metabolite-KYNA in the plasma and tissue of studied animals, whereas KYN, the KYN/TRP ratio and QUIN were not affected by 1-MT. Moreover, 1-MT did not inhibit the LPS-induced degradation of TRP to KYN, albeit the increase in KYNA indicated degradation to one branch of the KP facilitated by 1-MT. Additionally, immunomodulatory effects were observed in the study, such as the suppression of LPS-induced maturation of neutrophils, reduced number of neutrophils and lower neutrophil to lymphocyte (N/L) ratio. In a cell culture of porcine lung fibroblasts, the authors showed that 1-MT increased CYP1A1 gene expression, which is a marker for AhR activation [164]. Previous research documented that 1-MT itself acts as an agonist for AhR [165], and the immunosuppressive effects of KYNA have been documented [166,167,168]. Wirthgen et al. [163] concluded that, despite there being no evidence for IDO1 inhibition in their study, the immunotolerogenic effects of 1-MT in LPS-induced sepsis could result from the interference of KYNA and 1-MT with AhR signalling. Agaugué et al. [169] examined the direct action of 1-MT on human DCs and showed that 1-MT had the ability to interfere with TLRs signalling in these cells and might modulate DCs function depending on the maturation signal but independently of its action on IDO1 activity.

However, LPS tolerance is not a universal occurrence but rather a mechanism of host defence that helps control the effects of inflammatory-mediated bacterial infection. The mice with endotoxin tolerance had increased numbers of Kupffer cells, neutrophils and an increase in phagocytosis of bacteria [170]. In pigeons, a reduction of leukocytes and eosinophils occurs together with an increase in lymphocytes [171]. Moreover, the endotoxin tolerance did not prevent the early TLRs signalling; nonetheless, it limited the later development of the inflammatory systemic disease.

**Figure 2:**
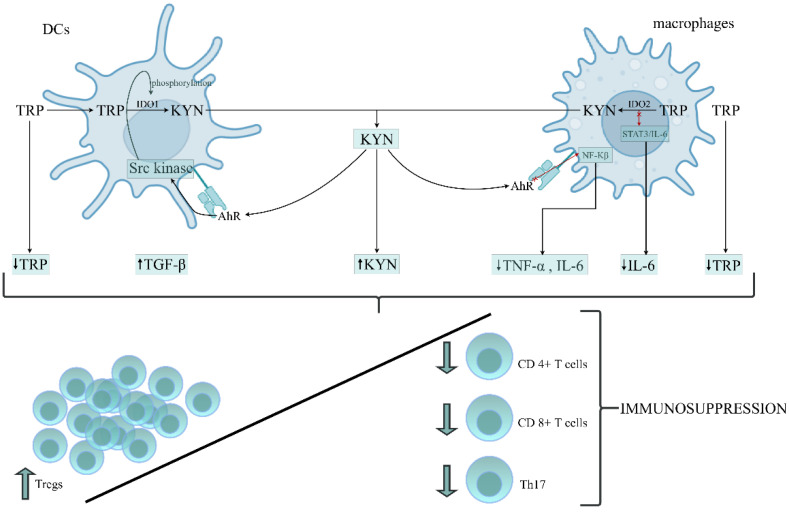
The proposed role of tryptophan (TRP) metabolism by the kynurenine pathway (KP) in the leading cells of innate immunity during the generation of immunosuppression/immunoparalysis during sepsis. DCs and macrophages belong to the APCs that mediate the cellular immune response by processing and presenting antigens for recognition by particular subsets of T cells. The activation of IDO1 and IDO2 in APCs initiates the metabolism of TRP via the kynurenine pathway, resulting in depletion of TRP and accumulation of kynurenine (KYN) in the immune cells’ environment. KYN, through AhR activation, can affect the different intracellular signalling pathways in DCs and macrophages, increasing the anti-inflammatory response and parallelly decreasing the proinflammatory response of these cells. Depletion of TRP and proinflammatory cytokines (IL-6, TNF-α), which is associated with the generation of KYN and anti-inflammatory cytokines, like TGF-β, creates an environment conducive to the origination of immunotolerogenic Tregs with a simultaneously reduced number and function of Th17, cytotoxic CD4+ and CD8+ T cells, leading to an overall impairment of immune functions and immunosuppression development. Abbreviations: DCs—dendritic cells; APCs—antygen presenting cells; IDO1—indoleamine 2,3-dioxygenase 1; IDO2—indoleamine 2,3-dioxygenase 2; KYN—kynurenine; AhR—aryl hydrocarbon receptor; NF-κB—the nuclear factor NF-kappaB; STAT3—the signal transducer and activator of transcription 3; IL-6—interleukin-6; TNF-α—tumor necrosis factor alpha; TGF-β—transforming growth factor-beta; Tregs—regulatory T cells; Th17—T helper 17.

The above data suggest that the role of IDO1 activation and its downstream metabolites in developing immunoparalysis during sepsis is not comprehensively resolved. Patients with a poor outcome presented enhanced IDO1 activity and increased levels of KYN, KYNA, TGF-𝛽 and IL-6 in the early stage of sepsis, resulting in the development of endotoxin tolerance. A rise in TGF-𝛽 and IL-6 levels on the first day of sepsis has been documented in nonsurvivors compared to survivors [172]. The occurrence of immunoparalysis was tightly related to a poor outcome in patients due to an imbalance between pro- and anti-inflammatory immune responses [173]. Therefore, the prolonged immunosuppressive phase prevents adequate control of primary or secondary infections with opportunistic pathogens [174]. Based on the above data, we constructed the hypothesis, proposing the role of TRP metabolism by KP in the leading cells of innate immunity in the formation of immunosuppression/immunoparalysis during sepsis (Figure 2).

## 7. Conclusions

Until now, published data on KP activation in SAKI was insufficient; therefore, defining its role in the disease remains unresolved. However, in light of the above-presented findings, IDO1 activation and generation of kynurenines play a pivotal immunomodulatory role in sepsis and septic shock. In the early stage of sepsis, the induction of IDO1 elicits powerful pro-inflammatory effects, which allows for fighting the invading pathogens. Simultaneously, excessive immune response could evoke severe tissue damage, leading to septic shock. On the other hand, IDO1 and KP activation attenuate immune reactions in host cells during ongoing sepsis, enabling the induction of immunotolerance, or even immunoparalysis, which contributes to the late morbidity and death due to incapability of developing an adaptive immunity against secondary opportunistic infections. IDO1 and KP metabolites appear to be part of the local immune regulatory mechanism in SAKI. Nevertheless, achieving a balance between antipathogenic activity and host defence against excessive immune response might be challenging in clinical practice.

## Data Availability

Not applicable.

## Abbreviations

1-MT	1-methyl-D-tryptophan
3-HKYN	3-hydroxykynurenine
AhR	aryl hydrocarbon receptor
AKI	acute kidney injury
AP-1	activator protein 1
APCs	antigen-presenting cells
ATP	adenosine triphosphate
BTK	Bruton’s tyrosine kinase
CCI	cecal content injection
CLP	cecal ligation, puncture
CpG-DNA	cytosine-phosphate-guanine dinucleotides present in bacterial DNA
CVVH	continuous veno-venous haemofiltration
CXCL1	C-X-C Motif Chemokine Ligand 1
CXCR2	C-X-C Motif Chemokine Receptor 2
DAMPs	damage-associated molecular patterns
DCs	dendritic cells
FLT3L	FMS-related tyrosine kinase 3 ligand
fMLP	N-formyl-methionyl-leucyl-phenylalanine
GCN2K	general control nonderepressible 2 kinases
GFR	glomerular filtration rate
GM-CSF	granulocyte-macrophage colony-stimulating factor
HMGB1	high-mobility group box 1
i.v.	intravenously
ICAM-1	intercellular adhesion molecule 1
ICU	intensive care unit
IDO1	indoleamine 2,3-dioxygenase 1
IDO2	indoleamine 2,3-dioxygenase 2
IFN-ɣ	interferon-gamma
IGFBP7	insulin-like growth factor-binding protein 7
IL-12	interleukin 12
IL-17A	interleukin-17A
IL-6	interleukin 6
IL-8	interleukin 8
iNOS	inducible nitric oxide synthase
IRAKM	IL-1 receptor-associated kinase M
ITK	IL-2 inducible T-cell kinase
KP	kynurenine pathway
KYN	kynurenine
KYNA	kynurenic acid
LPS	lipopolysaccharide
Mac-1	macrophage-1 antigen
MCP-1	monocyte chemoattractant protein-1
MIF	migration inhibitory factor
MPO	myeloperoxidase
mtDNA	mitochondrial DNA
mTOR	mammalian target of rapamycin
MyD88	Myeloid Differentiation Factor 88
NETs	neutrophil extracellular traps
NFAT	nuclear factor of activated T cells
NF-κB	nuclear factor kappa-light-chain-enhancer of activated B cells
NGAL	neutrophil gelatinase-associated lipocalin
NK	natural killer
NLRs	nucleotide-binding oligomerization domain-like receptors
NOX-2	neutrophils possess enzymes-NADPH oxidase
PAMPs	pathogen-associated molecular patterns
PDL1	programmed death-ligand 1
PPAR-α	peroxisome proliferator-activated receptor α
QUIN	quinolinic acid
ROS	reactive oxygen species
RTECs	renal tubular epithelial cells
SAKI	sepsis-induced AKI
SIRS	systemic inflammatory response syndrome
STAT3	signal transducer and activator of transcription 3
Syk	spleen tyrosine kinase
TDO2	tryptophan 2,3-dioxygenase 2
TGF-β	transforming growth factor β
Th17	T helper 17
Th2	T helper 2
TIMP-2	tissue inhibitor of metalloproteinase-2
TLR4	Toll-like receptor 4
TLRs	Toll-like receptors
TNF-α	tumor necrosis factor α
Tregs	regulatory T cells
TRP	trypthofan
VCAM-1	vascular cell adhesion molecule 1
WT	Wild-type mice
